# The Role of p38 Mitogen-Activated Protein Kinase-Mediated F-Actin in the Acupuncture-Induced Mitigation of Inflammatory Pain in Arthritic Rats

**DOI:** 10.3390/brainsci14040380

**Published:** 2024-04-14

**Authors:** Xu Zhou, Yu-Chen Zhang, Kai-Qiu Lu, Ran Xiao, Wen-Chao Tang, Fan Wang

**Affiliations:** School of Acupuncture-Moxibustion and Tuina, Shanghai University of Traditional Chinese Medicine, Shanghai 201203, China; chrysalis@alumni.sjtu.edu.cn (X.Z.); process@alumni.sjtu.edu.cn (Y.-C.Z.); lucaslu@alumni.sjtu.edu.cn (K.-Q.L.); duolabmeng@alumni.sjtu.edu.cn (R.X.); vincent.tang@shutcm.edu.cn (W.-C.T.)

**Keywords:** acupuncture, p38 MAPK pathway, inflammatory pain, acupoint area

## Abstract

The analgesic efficacy of acupuncture has been widely recognized. However, the mechanism by which manual acupuncture-generated mechanical stimuli translate into biological signals remains unclear. This study employed a CFA-induced inflammatory pain rat model. Acupuncture intervention was then performed following standardized procedures. Enzyme-linked immunosorbent assay (ELISA) assessed inflammatory cytokines levels, while immunofluorescence and qRT-PCR screened the level of p38 and F-actin expression in the ST36 acupoint area of rats. Results indicated increased inflammatory factors, including IL-1β and TNFα, with reduced paw withdrawal mechanical threshold (PWMT) and paw withdrawal thermal latency (PWTL) in CFA rats compared to unmodeled rats. After acupuncture intervention, the heightened expression level of F-actin and p38 mRNA and the phosphorylation of p38 in the acupoint area was observed alongside decreased inflammatory factors in diseased ankle joints. The application of lifting and thrusting manipulations further enhanced the effect of acupuncture, in which the molecular expression level of muscle and connective tissue increased most significantly, indicating that these two tissues play a major role in the transformation of acupuncture stimulation. Moreover, antagonizing p38 expression hindered acupuncture efficacy, supporting the hypothesis that p38 MAPK-mediated F-actin transduces mechanical signals generated by acupuncture and related manipulation into biological signals.

## 1. Introduction

Acupuncture is a therapy involving the insertion of fine needles into specific body areas to exert therapeutic effects by correcting energy imbalances [1]. Thought to have therapeutic effects on over 100 kinds of diseases, acupuncture has been most widely studied for its efficacy on pain [2]. Specifically, acupuncture is effective in relieving inflammatory pain [3], neuropathic pain [4,5], and cancer pain [6]. Therefore, acupuncture is recommended by several guidelines as an essential complementary and alternative therapy for pain relief [7,8,9].

Manual acupuncture is a kind of method that utilizes specific needle manipulation techniques, such as lifting, thrusting, and rotating, to stimulate the local area and achieve certain therapeutic effects [10]. Studies have found that different acupuncture techniques can change blood flow perfusion, electrical signals in the spinal dorsal horn, and skin temperature to exert different regulatory effects [11,12]. Unlike the electroacupuncture technique, which is mainly based on electrical stimulation, manual acupuncture is pure mechanical stimulation for the organism, which must be converted into biological signals to be recognized and transmitted by the organism [13]. Thus, exploring the process of converting the mechanical stress generated by hand needling into intracellular biological signals is the primary issue in studying acupuncture manipulation’s effect mechanism and law of action.

Anatomically, most acupoints are located in areas with densely distributed inherent connective tissue [14]. As the most widespread cells in connective tissue, fibroblasts are connective tissue’s major sensory and effector cells [15]. Our previous research showed that fibroblasts are vital cells in the connective tissue of the acupoint area in response to the stimulation of manipulation. The application of acupuncture manipulation can elongate the morphology of fibroblasts in the acupoint area more significantly and, at the same time, promote the secretion of signaling molecules, such as adenosine triphosphate (ATP) [16]. Present studies have shown that ATP released by mechanical stimulation can activate purinergic receptors on nearby fibroblasts and lead to an intracellular mitogen-activated protein kinase (MAPK) cascade [17,18]. This biological process mediates the remodeling and proliferation of the fibroblast cytoskeletal microfilament protein F-actin and is crucial in converting mechanical to biochemical signals [19]. Among them, p38 MAPK, a member of the MAPK family, is an important pathway mediating cellular force transduction. It can be activated by extracellular mechanical stress or stretch to participate in physiological and pathological processes such as cytoskeletal remodeling, proliferation, and differentiation [20,21]. However, the role of p38 MAPK in the mechanical signaling mechanism of acupuncture needs to be further investigated.

Therefore, this study aimed to confirm that p38 MAPK in the acupoint area is the critical signal converting mechanical signals to biological signals from acupuncture. In our study, we applied a rat model of acute inflammatory pain and intervened by acupuncture with lifting and thrusting manipulations on the ST36 acupoint of the affected foot. By observing the effects of acupuncture on the phosphorylation level of p38 MAPK (p-p38) and the expression levels of F-actin in acupoint fibroblasts, the role of p38 MAPK in the acupuncture initiation mechanism within the acupoint area fibroblasts was investigated. 

## 2. Materials and Methods

### 2.1. Animals

Adult male Sprague Dawley rats (180–300 g) were sourced from the Experimental Animal Center of Shanghai University of TCM, with the animal license number SYXK (Shanghai) 2020-0009. All rat handling and experimental procedures adhered to the Guidelines for ethical review of animal welfare (GB/T 35892-2018). This study was approved by the laboratory animal ethics committee of Shanghai University of TCM (No. PZSHUTCM220613016).

### 2.2. Environmental Adaptation and Grouping

All rats were housed in a temperature-controlled environment with a 12-12 h light–dark cycle, provided with food and water ad libitum. After a 7-day adaptive period, 40 rats were randomly allocated into five groups (8 rats per group):

- Group I (control group): Normal rats with gripping and holding only;
- Group II (CFA group): Arthritic rats with gripping and holding only;
- Group III (MA group): Arthritic rats treated with minimum acupuncture without lifting and thrusting manipulations;
- Group IV (LTM group): Arthritic rats treated with acupuncture lifting and thrusting manipulations;
- Group V (LTM + p38^−^ group): Arthritic rats treated with acupuncture manipulations simultaneously with p38 antagonist injection.

### 2.3. Establishment of Arthritic Rat Model

**Figure 1:**
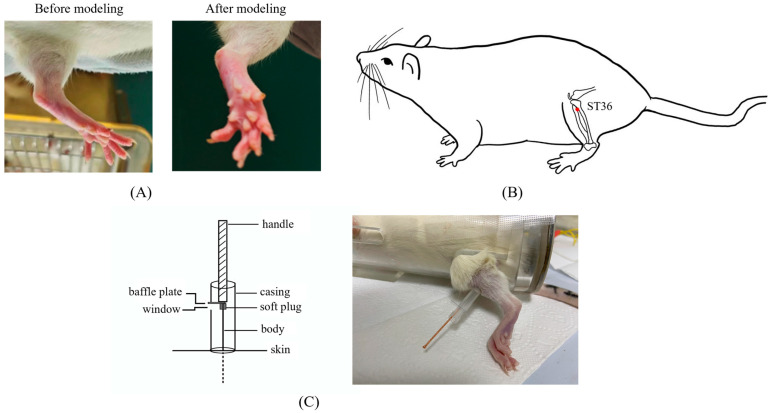
The details of modeling and acupuncture treatment. (**A**) Swollen left hindfoot metatarsophalangeal joint one day after modeling. (**B**) Location of the acupuncture point ST36 used in this study. (**C**) The self-made acupuncture needle cannula used to control the amplitude of the lifting and thrusting manipulations.

Arthritis was induced in rats, excluding the eight in the control group, by subcutaneously injecting 0.1 mL of 10mg/mL complete Freund’s adjuvant (CFA) into the center of their left hind paws. The peak of foot swelling (acute inflammatory response) occurred at 18–27 h after CFA injection [22] (Figure 1A). The remaining eight rats were injected with equal doses of physiological saline. Combined with pre-experimental experience, acupuncture treatment began one day after the model was established to address inflammation. CFA was obtained from Sigma Chemical Co. (St. Louis, MO, USA, F5881). 

### 2.4. Acupuncture Treatment

To facilitate smooth acupuncture, rats underwent the soft cloth fixation method. Disposable acupuncture needles (0.25 × 13 mm, Hwato, Suzhou Medical Appliance Factory, Suzhou, China) were vertically inserted into a 7 mm depth in the left ST36, located on the posterior lateral aspect of the knee, approximately 5 mm below the fibular tuberosity [23] (Figure 1B). The needle position was adjusted if signs of nerve or blood vessel irritation were noted.

After achieving *deqi*, the practitioner felt tenseness around the needle [24]. For the MA group, needles were removed after 30 min of maintenance without manipulation. In LTM and LTM + p38^−^ groups, needles were lifted and thrusted with an amplitude of about 2 mm and a frequency of 60 cycles per minute. During each acupuncture session, there was a 1 min period of lifting and thrusting, followed by a 9 min interval, totaling 30 min per session. Acupuncture was administered by a skilled acupuncturist once a day for seven days. A self-made acupuncture needle cannula (Figure 1C) and a metronome were used to control amplitude and frequency, respectively [25]. Rats in the control and CFA groups underwent the same fixation method as experimental groups without additional interventions.

### 2.5. p38 Antagonist Injection

The p38 MAPK inhibitor SB203580 obtained from MedChemExpress (Shanghai, China, HY-10256) was diluted and administered intramuscularly at a dose of 15 mg per kg. This dosage was determined via a gradient pre-experiment, wherein rats exhibited the lowest levels of p38 mRNA expression in the ST36 acupoint area while maintaining normal physical signs, including diet, defecation, and coat color (Table S1). The administration took place for seven consecutive days following the baseline pain threshold measurement and continued during the initial six days of the intervention.

### 2.6. Behavioral Testing

The manual von Frey test, considered to be the gold standard for assessing mechanical thresholds [26], was employed to evaluate the paw withdrawal mechanical threshold (PWMT). During measurements, rats were placed in small cages with a mesh. Von Frey filaments of various forces (2, 4, 6, 8, 10, 15, 26, and 60 g, North Coast Medical, Inc., Morgan Hill, USA) were applied perpendicularly to the plantar surface of the left hind paw until they buckled, maintained for 2–5 s. The PWMT was determined as the lowest force that promoted paw withdrawal at least twice in 5 applications [27].

The hot plate test assessed paw withdrawal thermal latency (PWTL). Unrestrained rats were positioned on a metal surface (YLS-6B, YuYan Instruments Ltd., Shanghai, China) maintained at (55.0 ± 0.5) °C, and the response latency was recorded via nocifensive behaviors like hind paw withdrawal or licking [28]. Each animal underwent testing three times at 15 min intervals, and the average of the three response times was considered the final PWTL. To prevent tissue damage, rats were removed from the hot plate after 60 s without observed nocifensive behaviors, with their PWTL recorded as 60 s [29].

In the initial stage, PWMT and PWTL were assessed before mold-making and systematic treatment to gauge the success of modeling. The second stage assessment was conducted at 1, 3, and 7 days post-acupuncture treatment.

### 2.7. ELISA Assay

IL-1β and TNF-α levels were quantified utilizing ELISA kits. The synovium of the ankle joint was extracted from the left hind paws of each rat group. Inflammatory factor levels in the tissue were measured from these focal ankle synovium samples, which underwent centrifugation at 3000 rpm for 10 min to separate the supernatants. The rat TNF-α and IL-1β ELISA kits were applied according to the manufacturer’s protocol (R&D Systems, Minneapolis, MN, USA, Cat# DY510/DY501). 

### 2.8. Total RNA Extraction and Quantitative Real-Time RT-PCR (qRT-PCR)

Rats were euthanized on the last day of acupuncture treatment after pain threshold measurement. Total RNA was isolated from tissue samples corresponding to the ST36 point, with dimensions of about 5 mm × 5 mm and a depth of approximately 8mm, using TRIzol reagent (Invitrogen, Carlsbad, USA Cat# 74104). The quality of the isolated RNA was measured using a Nano One spectrophotometer (Eppendorf, Hamburg, Germany, Biopgotometer plus), configured to measure absorbances at 260 and 280 nm. The 260/280 absorbance ratios falling within the range of 1.8 to 2.0 indicated that the RNA was free of contamination (Table S2) [30]. Additional singleplex qPCR for p38 and β-actin ensured that the samples were not contaminated with genomic DNA (Table S3, Figure S1). For cDNA preparation, two µg was reverse-transcribed using Oligo (dT) 18 primers (Invitrogen) and M-MLV Reverse Transcriptase (Promega Co., Ltd., Madison, USA, Cat# M1701), following the manufacturer’s instructions. qRT-PCR was performed to assess p38 expression using SYBR green (TaKaRa Biomedical Technology (Beijing) Co., Ltd. Beijing, China, Cat# DRR081A) on an Applied Biosystems ViiA™ 7 (ABI, London, USA) under the following conditions: 95 °C for 1 min, followed by 40 cycles of 95 °C for 15 s and 60 °C for 30 s. Each reaction was conducted in triplicate, and the mean of at least three experiments was calculated. All results were normalized to β-actin and determined using the relative (2^−ΔΔCt^) quantification method. Primers used in the study are shown in Table 1.

### 2.9. Immunofluorescence

The skin, subcutaneous connective, and muscle tissues of the ST36 area were collected and fixed, and paraffin sections were prepared. Slices were deparaffinized in xylene and rehydrated with ethanol. Antigen retrieval was performed by microwave treatment of slices in EDTA Antigen Retrieval Solution (pH 9.0). The cells were treated with PBS (pH 7.4) and 3% BSA (Beijing Solarbio Science & Technology Co., Ltd., Beijing, China, Cat# A8020), followed by incubation with the primary antibody (anti-p38 [1:100, Proteintech] or anti-F-actin [1:400, Proteintech]) at 4 °C overnight. After washing, goat anti-mouse RedX secondary antibody (1:400) was added, and samples were incubated in the dark for 45 min at 37 °C. Cells were washed with PBS and counterstained with DAPI, followed by spontaneous fluorescence quenching reagent and the application of a coverslip [31]. PBS was incubated as a negative control to ensure the specific binding of antibodies to the target proteins in immunofluorescence (Figure S2). The slices were observed and photographed under a fluorescence microscope (OLYMPUS CX53 & DP70, OLYMPUS, Tokyo, Japan). Fluorescence quantification was performed using DP2-BSW application software (Version 2.2, OLYMPUS, Japan).

### 2.10. Statistical Analysis

All data in this study are presented as mean  ±  standard deviation. The Shapiro–Wilk test was used to determine normality for each variable, and Levene’s test was used to assess homogeneity of variance. Independent t and Mann–Whitney U tests were utilized for comparisons between the two groups. One-way analysis of variance (ANOVA) and Kruskal–Wallis tests were applied for comparisons among ≥three groups. Post hoc tests involved the Least square difference (LSD) and Bonferroni correction for multiple tests. Data from repeated measurements were analyzed using repeated measurements ANOVA and Scheirer–Ray–Hare test. Spearman’s correlations were used to assess correlations between the expression of F-actin and p-p38 in fibroblasts. Statistical analyses were performed using SPSS Statistics, version 24.0 (IBM Corp., Armonk, NY, USA). Differences with *p* < 0.05 were considered statistically significant. Preliminary analysis indicated that at least six rats were needed at each time point and in each group to achieve 80% efficacy (1-β) and 90% confidence [32].

## 3. Results

### 3.1. Effects of Acupuncture on Pain Threshold

**Figure 2:**
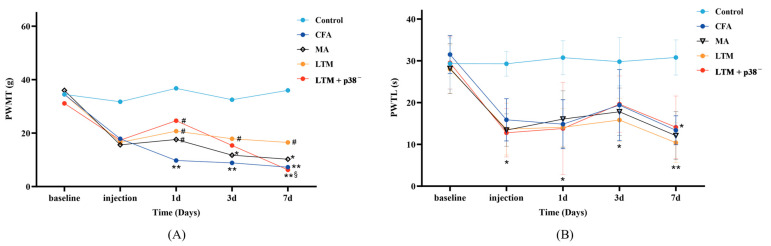
Effects of acupuncture on nociceptive behaviors in the CFA-induced rat arthritic model. (**A**) PWMT and (**B**) PWTL were evaluated 24 h before CFA injection (baseline), 1 day after CFA injection (injection), and 1, 3, and 7 days after acupuncture treatment. PWMT, the paw withdrawal mechanical threshold; PWTL, the paw withdrawal thermal latency. * *p* < 0.05 and ** *p* < 0.001 compared with the control group; # *p* < 0.05 compared with the CFA group; § *p* < 0.05 compared with the LTM group. *N* = 8 in each group. PWMT, paw withdrawal mechanical threshold; PWTL, paw withdrawal thermal latency; CFA, complete Freund’s adjuvant; MA, minimum acupuncture; LTM, lifting and thrusting manipulations.

Nociceptive behaviors were assessed via the observation of PWMT (Figure 2A) and PWTL (Figure 2B). Initially, no significant differences in either PWMT or PWTL were observed (*p* > 0.05). After modeling, in comparison with the control group, rats in other groups demonstrated a decreasing trend in both behavioral tests, with PWTL being more significant (*p* < 0.05), indicating a successful establishment of the rat arthritic model.

According to Table 2, the PWMT was not significantly affected by the interaction effect (“Intervention” × “Time”) [H = 5.677, *p* = 0.991]. The main effects of “Intervention” [H = 9.581, *p* < 0.048] and “Time” [H = 11.361, *p* = 0.023] on PWMT were significant. Pairwise comparisons revealed a significant increase (*p* < 0.05) in PWMT among the three intervention groups compared to the CFA group as soon as acupuncture commenced for one day. Throughout the seven-day treatment, lifting and thrusting manipulations led to a noteworthy increase in PWMT (*p* < 0.05) compared with the CFA group and surpassed the PWMT score of the MA group, suggesting that acupuncture manipulation may alleviate pain in rats with inflammation more effectively than needle retention. According to Table 3, the PWTL was significantly affected by the interaction effect (“Intervention” × “Time”) [F = 3.040, *p* < 0.001], along with the simple effects of “Intervention” [F = 38.753, *p* < 0.001] and “Time” [F = 24.368, *p* < 0.001]. However, there were no statistically significant differences in PWTL during treatment among the four groups except for a notable decrease compared to the control group (*p* < 0.05 or *p* < 0.001).

### 3.2. Effects of Acupuncture on the Expression of Localized Inflammatory Factors

**Figure 3:**
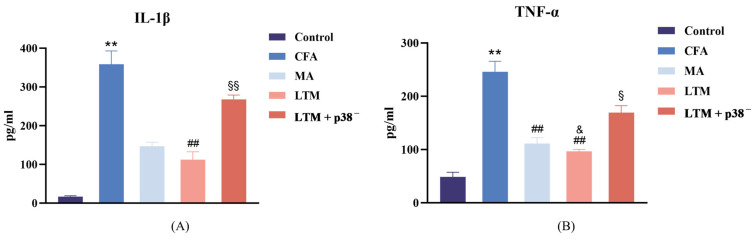
(**A**) Interleukin-1 beta (IL-1β) and (**B**) tumor necrosis factor-alpha (TNF-α) levels in the inflamed ankle joint of CFA-induced rat arthritic model after a seven-day intervention. Data are shown as mean ± SD. ** *p* < 0.001 compared with the control group; ## *p* < 0.001 compared with the CFA group; & *p* < 0.05 compared with the MA group; § *p* < 0.05 and §§ *p* < 0.001 compared with the LTM group. *N* = 8 in each group. CFA, complete Freund’s adjuvant; MA, minimum acupuncture; LTM, lifting and thrusting manipulations.

Following drug injection for modeling, the concentration of IL-1β (Figure 3A) and TNF-α (Figure 3B) in the focal tissues of the CFA group increased (*p* < 0.001). Compared to the CFA group, the LTM group exhibited a significant decrease in IL-1β levels in the focal ankle synovium (*p* < 0.001), while both the MA group and LTM group showed a significant decrease in TNF-α levels (*p* < 0.001). Furthermore, the reduction in TNF-α in the LTM group exceeded that of the MA group (*p* < 0.05). Although a decrease in IL-1β was also observed in the MA group, it did not reach statistical significance (*p* = 0.054). These findings suggest that acupuncture can diminish the expression of inflammatory factors in the focal tissues of rats with inflammatory pain, with the application of lifting and thrusting manipulations intensifying this effect. Additionally, IL-1β and TNF-α levels in the LTM+p38^−^ group displayed a significant increase (*p* < 0.001 and *p* < 0.05, respectively) compared with the LTM group, indicating a blockade of the needling effect by the p38 antagonist.

### 3.3. Effects of Acupuncture on p38 mRNA Expression in the Acupoint Area

**Figure 4:**
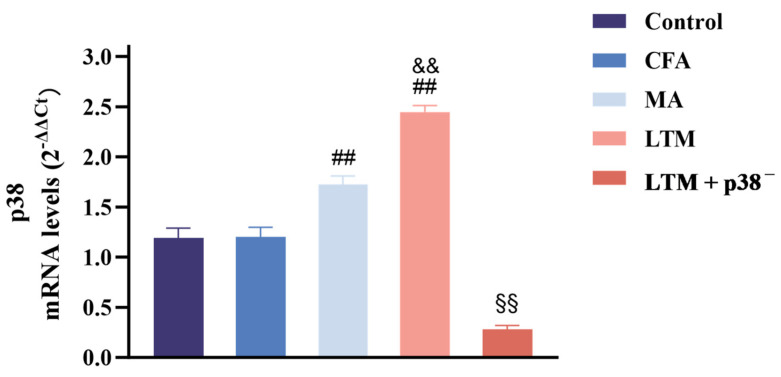
Real-time PCR analysis of p38 mRNA expression in the ST36 acupoint area of the CFA-induced rat arthritic model after a seven-day intervention. Relative quantification was performed using the comparative Ct method (2^–ΔΔCt^). Results are expressed as arbitrary units (control = 1) and presented as mean ± SD. ## *p* < 0.001 compared with the CFA group; && *p* < 0.001 compared with the MA group; §§ *p* < 0.001 compared with the LTM group. *N* = 8 in each group. CFA, complete Freund’s adjuvant; MA, minimum acupuncture; LTM, lifting and thrusting manipulations.

In Figure 4, no significant change (*p* > 0.05) was noted between the control and CFA groups regarding p38 mRNA expression in the acupoint area. However, following acupuncture treatment, p38 expressions increased significantly in both cases (*p* < 0.001) compared to the CFA group, with the LTM group exhibiting a more pronounced elevation (*p* < 0.001). This suggests that acupuncture can enhance the expression of p38 mRNA in the acupoint area of rats, and acupuncture manipulation intensifies this effect. Subsequent p38 antagonist injection resulted in a significant reduction (*p* < 0.001) in p38 expression in tissues corresponding to ST36 of rats, indicating successful blockade of the p38 MAPK pathway.

### 3.4. Effects of Acupuncture on the Expression of F-Actin and p-p38 in Fibroblasts of the Acupoint Area

**Figure 5:**
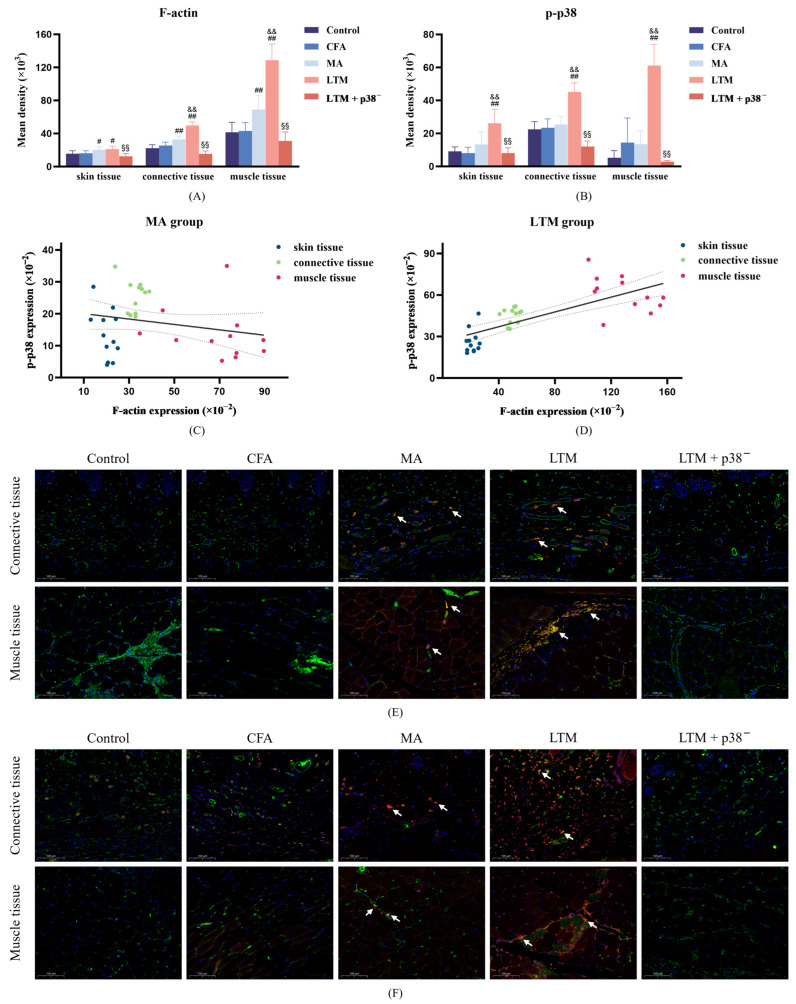
F-actin expression and p-p38 level in fibroblasts of the ST36 acupoint area after a seven-day intervention. (**A**,**B**) Fluorescence intensity of F-actin filaments and p-p38 in fibroblasts from skin, connective, and muscle tissues. # *p* < 0.05 and ## *p* < 0.001 compared with the CFA group; && *p* < 0.001 compared with the MA group; §§ *p* < 0.001 compared with the LTM group. *N* = 12 in each group. (**C**,**D**) The linear correlation between F-actin expression and p-p38 level in the MA and LTM groups. (**E**,**F**) Immunofluorescence staining for (**E**) F-actin and (**F**) p-p38 (red), Vimentin (a fibroblast marker) (green), and DAPI (blue) in fibroblasts of the acupoint area with different interventions. The orange and yellow zones (indicated by arrows) denote the colocalization of all three elements within the loose subcutaneous connective tissue and intermuscular connective tissue in both the MA and LTM groups. Therefore, fibroblasts expressing F-actin or p-p38 positively are predominantly distributed in these regions. Scale bar, 40×: 100 μm. p-p38, phosphorylated p38 MAPK; CFA, complete Freund’s adjuvant; MA, minimum acupuncture; LTM, lifting and thrusting manipulations.

Neither F-actin expression (Figure 5A) nor p-p38 level (Figure 5B) in fibroblasts of skin, subcutaneous connective, and muscle tissues in the acupoint area showed significant differences from the control group (*p* > 0.05) after modeling. However, compared with the CFA group, F-actin expression in the MA group was significantly enhanced in all sampled tissues (*p* < 0.05 or *p* < 0.001), while the p-p38 level exhibited no significant changes in the acupoint area. Furthermore, via lifting and thrusting manipulations, F-actin expressions in the subcutaneous connective and muscle tissues were significantly enhanced (*p* < 0.001), and the level of p-p38 at all sampled tissues was significantly increased (*p* < 0.001) compared with needle retention. This indicates that acupuncture can increase the expression of F-actin and p-p38 in acupoint area fibroblasts of rats, with acupuncture manipulation intensifying this effect. After the p38 antagonist injection, F-actin and p-p38 levels were significantly weakened compared with the LTM group (*p* < 0.001). Additionally, a positive linear correlation in the LTM group confirmed the relationship between p-p38 and corresponding F-actin expression (r = 0.740, *p* < 0.001; Figure 5D). Therefore, applying acupuncture manipulation might be a more efficient way to activate the p38 signaling pathway (Figure 5F) to induce higher expression of F-actin (Figure 5E) (Figure S3).

## 4. Discussion

Analgesia stands as a cornerstone in acupuncture therapy, dating back thousands of years to ancient China when physicians began utilizing it to relieve different types of pain [33]. Thus, the pain model becomes an effective vehicle for exploring the mechanical signaling pathways of acupuncture. According to traditional Chinese Medicine theory, the principal pathology underlying pain involves the blockage and lack of blood flow and qi. ST36, as the *he*-sea point of the stomach meridian of foot Yangming, holds the capability of dredging meridians and harmonizing qi and blood [34]. Literature reviews have confirmed ST36 as one of the most frequently used acupoints for treating inflammatory pain [3,35]. Therefore, in this study, the CFA model of inflammatory pain was selected as the target for acupuncture intervention, with focus directed towards the acupoint “ST36”. Results from this study demonstrate that acupuncture treatment at ST36 effectively alleviated pain and mitigated the expression of localized inflammatory factors in rats with CFA.

Acupuncture analgesia is inextricably linked to the neural system. The detection of painful stimuli initiates with the activation of peripheral sensory neurons known as nociceptors. The inflammatory substances induced by CFA can sensitize nociceptors, enhance neuronal excitability, reduce pain thresholds, and ultimately induce pain sensations [36]. In the present study, a significant increase in PWMT after intervention indicated that acupuncture effectively improved mechanical nociceptive hypersensitivity in CFA rats. However, no significant change was observed in PWTL after acupuncture treatment. Considering that sensory nerve cell bodies innervating acupuncture points are clustered in the dorsal root ganglion (DRG) [37], the disparity in these outcomes may be attributed to the involvement of DRG neurons expressing the Transient Receptor Potential Vanilloid 1 (TRPV1). TRPV1, an ion channel expressed by peripheral nociceptors, is activated by heat and modulated by inflammatory mediators [38]. A previous study [39] reported that TRPV1-expressing DRG neurons, primarily responsible for mediating PWTL, are not implicated in acupuncture analgesia at the ST36 acupoint. Instead, they appear to facilitate the pain sensation of the hind paw.

Mitogen-activated protein kinases (MAPKs) are a class of serine-threonine protein kinases extensively distributed across eukaryotic cells. Among them, p38 MAPK emerges as a key signaling pathway in fibroblasts, responding to mechanical stimuli [40]. It plays a crucial role in mechanical stress-induced cytoskeletal reorganization [21] and the regulation of inflammation [41]. In this study, heightened p38 expression was observed across all experimental groups following acupuncture intervention, compared to the CFA group. Particularly significant was the elevated p38 expression in the LTM group. This trend was associated with a decrease in the expression of local inflammatory factors (e.g., IL-1β and TNF-α). IL-1β and TNF-α have potent pro-inflammatory activities that promote the secretion of various pro-inflammatory mediators [42]. Upon inhibition of p38 expression, the impact of acupuncture manipulation on inflammatory factors in the focal tissues of modeled rats was attenuated. This suggests a correlation between p38 MAPK expression in acupoint tissue and the effectiveness of acupuncture manipulation. P38 MAPK is generally recognized to be activated by inflammatory responses, exhibiting potent and complex pro-inflammatory effects [43]. However, recent studies [44,45] have suggested a potential anti-inflammatory role for this signaling pathway. Specifically, the activation of mitogen- and stress-activated kinases (MSKs) by p38α or by ERK1 and ERK2 leads to increased transcription of the anti-inflammatory cytokines interleukin-10 and IL-1 receptor antagonist [46,47]. The determining mechanisms involved still need to be confirmed by further studies.

The fascia tissue in the connective tissue of the acupoint area comprises three levels from superficial to deep: dermal dense connective tissue, loose subcutaneous connective tissue, and intermuscular connective tissue. To determine whether fibroblasts at all levels of the fascia participate in acupuncture signal transduction by activating p38 MAPK, immunofluorescence histochemical assay techniques were further applied to observe the expression and distribution of p-p38 and F-actin in fibroblasts within connective tissues at different depths of the acupoint area. Results revealed that compared with the MA group, the level of p-p38 in the skin, subcutaneous connective tissue, and muscle tissue of the LTM group increased by 95.24%, 77.86%, and 354.01%, respectively, while F-actin increased by 4.57%, 20.69%, and 86.74%, respectively. Furthermore, both p-p38 and F-actin exhibited the highest absolute values in muscle tissue, and fibroblasts with positive expression of p-p38 and F-actin were mainly distributed in the intermuscular connective tissue. A previous study using a purine agonist to mimic pinprick stimulation induced transient changes in fibroblast F-actin and caused cellular remodeling [48]. When dynamic structural changes are initiated, signaling and physical factors induce quiescent fibroblasts to form myofibroblasts. These activated myofibroblasts can modulate resident immune cell functions, thereby controlling inflammation development [49]. Therefore, the activation of myofibroblasts by structural changes in the acupoint area caused by acupuncture manipulation may be the biological basis for their anti-inflammatory effect. These experimental results indicate that connective tissue within the muscle may be the primary structure translating the effects of acupuncture, with muscle playing a more critical role in responding to stimuli from acupuncture manipulation.

F-actin, an integral cytoskeleton component, allows externally applied mechanical forces to be transmitted to the cell and translated into biochemical responses [50]. As the bridge connecting two critical nodes in the cellular perception of mechanical stimuli, the regulatory relationship between p38 MAPK and F-actin has been extensively explored. An experiment revealed that mechanical stretch can activate MAPK and induce reinforcement of F-actin stress fibers [19]. Further investigation identified p38 MAPK /MAPK-activated protein kinase 2 (MK2)/heat shock protein 27 (Hsp27) as the major signaling pathway regulating F-actin dynamics [51]. In this study, the increased p-p38 level induced by acupuncture positively correlated with the expression level of F-actin, whereas antagonizing p38 expression suppressed the high expression of F-actin in the tissues of acupoint areas stimulated by acupuncture manipulation. These results suggest that p38 MAPK mediates the acupuncture-induced proliferation of F-actin in the acupoint area, with this regulatory relationship further reinforced after acupuncture manipulation. However, the changing characteristics of F-actin and p-p38 were not completely consistent. While F-actin was significantly increased in fibroblasts of all tissue samples in the MA group, p-p38 was not significantly increased, despite a significant increase in p38 mRNA in the acupoint tissues. In addition, acupuncture manipulation significantly increased the p-p38 level in fibroblasts of skin tissue, but the change in F-actin was insignificant. These inconsistent results underscore the complexity of the acupuncture initiation mechanism. Specifically, the activated p38 signaling pathway is not exclusive to fibroblasts, and p38 is not the only signaling pathway capable of influencing F-actin expression [52].

This study, for the first time, demonstrates that lifting and thrusting manipulations can activate the p38 MAPK signaling pathway of fibroblasts in the acupoint area. This activation alters the expression and distribution of the microfilament protein F-actin in the fibroblast cytoskeleton and induces remodeling of the fibroblast cytoskeleton. Consequently, the mechanical stress of acupuncture is transduced into biochemical signals, thereby initiating the analgesic effect of acupuncture. These results establish a new experimental foundation for exploring the biomechanical transduction mechanism of acupuncture manipulation. 

Nevertheless, it is essential to acknowledge the limitations of our study. Firstly, while our experiment suggests that the F-actin protein may participate in the transduction of mechanical force signals to biochemical signals from acupuncture, the absence of a reference group blocking the expression of the F-actin protein makes it difficult to ascertain whether F-actin is the primary or sole link in the signal transduction mechanism observed. Further experiments are necessary to verify these speculations. Secondly, considering that acupuncture exhibits various therapeutic effects beyond analgesia and anti-inflammatory action and recognizing that the CFA model may not encompass all pathophysiologic states, additional animal models should be incorporated. This approach allows for the replication of findings and facilitates a deeper exploration of the comprehensive mechanisms of acupuncture effects. Furthermore, while fibroblasts are an important component of connective tissue, they are not the sole constituents. Other components in connective tissue, such as mast cells, macrophages, and the blood vessels and nerves, also play vital roles in the initiation of the needling effect [53]. Investigating how p38-induced fibroblast remodeling interacts with these tissues and cells to amplify local needling information and deliver it to the lesion will be our main research focus in the future.

## 5. Conclusions

In summary, our results show that the levels of pro-inflammatory factors IL-1β and TNF-α were increased in rats with inflammatory pain. After acupuncture intervention, activation of the p38 MAPK signaling pathway in the acupoint area initiated the analgesic effect by promoting F-actin proliferation and structural remodeling. Moreover, the application of lifting and thrusting manipulations further enhanced acupuncture efficacy. During the conversion of acupuncture mechanical stimuli to biological signals, intermuscular connective tissue emerged as the primary structure translating the acupuncture effect. These findings suggest that the p38 MAPK-mediated F-actin mediates the biological response of fibroblasts in the acupoint area to manual acupuncture stimulation.

## Figures and Tables

**Table 1:** Primers for quantitative real-time PCR.

Name	Accession No	Amplicon Size (bp)	Sequence (5′–3′)
rat p38	NM_031020.2	92	Forward: GGT GTG TGC TGC TTT TGA TAC Reverse: TCC TTT TGG CGT GAA TGA
rat β-actin	NM_031144.3	155	Forward: CCT CTA TGC CAA CAC AGT Reverse: AGC CAC CAA TCC ACA CAG

**Table 2:** Scheirer–Ray–Hare test of PWMT.

Variation	SS	df	MS	H	*p*-Value
Intervention	126,910.188	4	31727.547	9.581	0.048
Time	150,485.038	4	37621.260	11.361	0.023
Intervention × Time	75,197.775	16	4699.861	5.677	0.991

SS, sum of squares of deviation; df, degree of freedom; MS, mean square.

**Table 3:** Repeated measurements ANOVA of PWTL.

Variation	SS	df	MS	F	*p*-Value
Intervention	4963.924	4	1240.981	38.753	<0.001
Time	4588.384	4	1147.096	24.368	<0.001
Intervention × Time	1476.109	16	92.257	3.040	<0.001

SS, sum of squares of deviation; df, degree of freedom; MS, mean square.

## Data Availability

The data contained within this article are available from the corresponding author, F.W., upon reasonable request. The data are not publicly available due to privacy.

## Supplementary Materials

The following supporting information can be downloaded at https://www.mdpi.com/article/10.3390/brainsci14040380/s1. Figure S1: The visualized results of singleplex qPCR for p38 and β-actin DNA; Figure S2: Immunofluorescence staining for the negative control; Figure S3: The immunofluorescence staining of skin tissue; Table S1: Gradient pre-experiment for p38 MAPK inhibitor SB203580 injection dosage; Table S2: The quality of the isolated RNA assessed by the 260/280 absorbance ratios; Table S3: Main parameters of singleplex PCR performed on p38 and β-actin.
