# Supplementary material for: The Role of p38 Mitogen-Activated Protein Kinase-Mediated F-Actin in the Acupuncture-Induced Mitigation of Inflammatory Pain in Arthritic Rats
Source: Brain Sci. 2024 Apr 14;14(4):380. doi: 10.3390/brainsci14040380 (PMC11048453; doi:10.3390/brainsci14040380)
Supplement: Supplementary file 1 [file brainsci-14-00380-s001.zip › Manuscript Figures-Supp.pdf]

Figure S1

The visualized results of singleplex qPCR for p38 and β-actin DNA

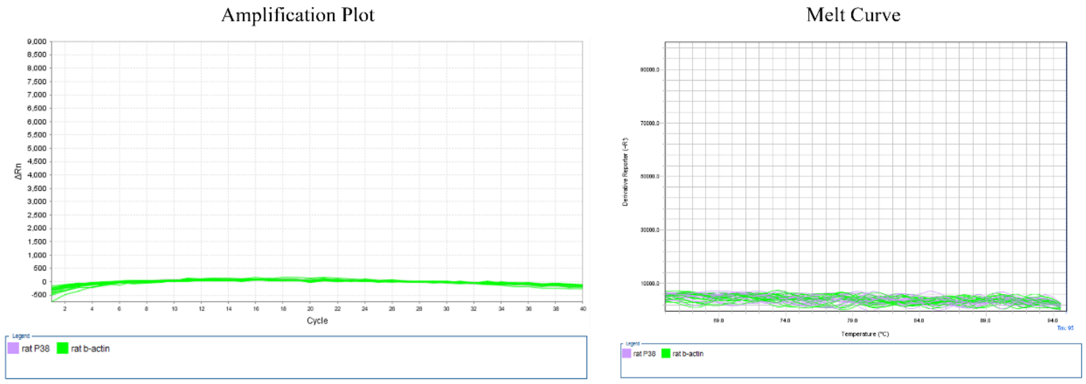

(A) (B)

Figure S1. The amplification plot (**A**) and melt curve (**B**) of singleplex qPCR for p38 and β-actin. Applying SYBR, a highly sensitive DNA fluorescent dye, the qPCR found no p38 or β-actin DNA was detected from the samples. The results indicated that the samples were not contaminated with genomic DNA thus further ruling out the possibility of false positives.

Figure S2

Immunofluorescence staining for the negative control

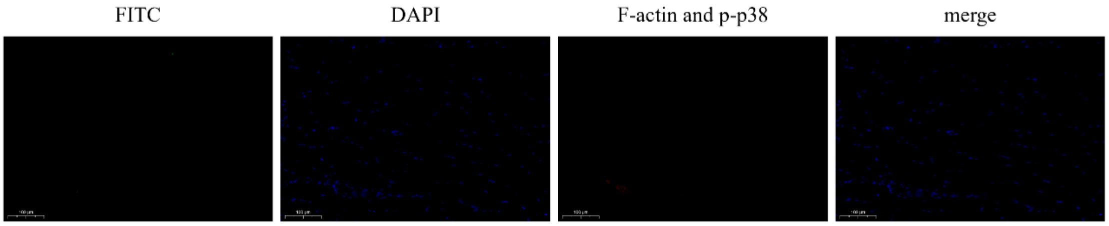

Figure S2. The negative control ensuring the specific binding of antibodies to the target proteins in immunofluorescence. PBS was incubated as a negative control for immunofluorescence. Scale bar, 40×: 100 μm. p-p38, phosphorylated p38 MAPK.

Figure S3

The immunofluorescence staining of skin tissue

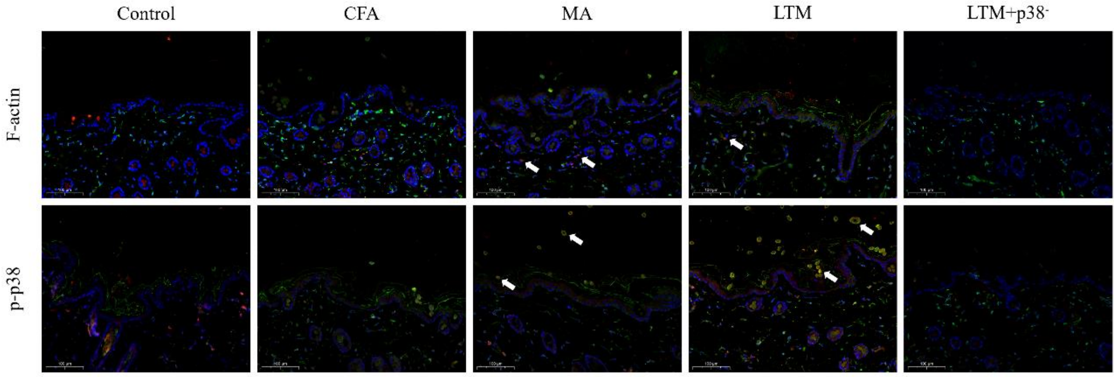

Figure S3. Immunofluorescence staining for F-actin and p-p38 (red), Vimentin (a fibroblast marker) (green), and DAPI (blue) in fibroblasts of the skin samples with different interventions. The orange and yellow zones (indicated by arrows) denote the colocalization of all three elements within the dermal dense connective tissue in both the MA and LTM groups. Scale bar, 40×: 100 μm. p-p38, phosphorylated p38 MAPK; CFA, complete Freund's adjuvant; MA, minimum acupuncture; LTM, lifting and thrusting manipulations.
