# Supplementary material for: The Role of p38 Mitogen-Activated Protein Kinase-Mediated F-Actin in the Acupuncture-Induced Mitigation of Inflammatory Pain in Arthritic Rats
Source: Brain Sci. 2024 Apr 14;14(4):380. doi: 10.3390/brainsci14040380 (PMC11048453; doi:10.3390/brainsci14040380)
Supplement: Supplementary file 1 [file brainsci-14-00380-s001.zip › Manuscript Tables-Supp.pdf]

Table S1

Gradient pre-experiment for p38 MAPK inhibitor SB203580 injection dosage

Sample No.	Gene	CT	Mean CT	ΔCT	$2^{-\Delta\Delta CT}$
normal	rat p38	26.024	25.967	10.528	6.770E-04
	rat p38	25.971			
	rat p38	25.905			
	rat GAPDH	15.481	15.438		
	rat GAPDH	15.392			
	rat GAPDH	15.441			
2.5	rat p38	25.333	25.432	10.449	7.153E-04
	rat p38	25.423			
	rat p38	25.541			
	rat GAPDH	15.004	14.983		
	rat GAPDH	14.983			
	rat GAPDH	14.962			
5	rat p38	26.446	26.416	10.603	6.431E-04
	rat p38	26.421			
	rat p38	26.382			
	rat GAPDH	15.858	15.814		
	rat GAPDH	15.770			
	rat GAPDH	15.813			
10	rat p38	26.535	26.484	10.946	5.069E-04
	rat p38	26.562			
	rat p38	26.354			
	rat GAPDH	15.605	15.537		
	rat GAPDH	15.495			
	rat GAPDH	15.512			
15	rat p38	26.269	26.281	11.626	3.165E-04
	rat p38	26.261			
	rat p38	26.313			
	rat GAPDH	14.689	14.655		
	rat GAPDH	14.650			
	rat GAPDH	14.627			
20	rat p38	28.453	28.460	13.762	7.201E-05
	rat p38	28.359			
	rat p38	28.568			
	rat GAPDH	14.794	14.699		
	rat GAPDH	14.637			
	rat GAPDH	14.665			

Table S1. The original data of the gradient pre-experiment for p38 MAPK inhibitor SB203580 injection dosage. Each reaction was conducted in triplicate. All results were normalized to GAPHD. GAPHD, glyceraldehyde-3-phosphate dehydrogenase; CT, cycle threshold values; ΔCT, gene Ct-β-actin Ct.

Table S2

The quality of the isolated RNA assessed by the 260/280 absorbance ratios

Sample No.	A260	A280	A260/280	RNA Concentration(ug/mL)
C1	0.486	0.263	1.85	972.9
C2	0.497	0.270	1.84	993.5
C3	0.568	0.302	1.88	1136.7
C4	0.577	0.306	1.89	1153.9
C5	0.549	0.288	1.91	1098.5
C7	0.559	0.293	1.91	1118.7
C8	0.517	0.271	1.91	1033.4
C21	0.524	0.278	1.88	1047.8
C22	0.603	0.328	1.84	1206.5
C9	0.600	0.324	1.85	1199.3
C10	0.572	0.304	1.88	1143.2
C11	0.566	0.302	1.87	1133
C12	0.492	0.271	1.82	983.7
C13	0.527	0.288	1.83	1055
C14	0.521	0.285	1.83	1042.7
C15	0.493	0.272	1.81	985
C16	0.502	0.276	1.82	1003.7
C17	0.536	0.294	1.82	1071.4
C18	0.542	0.297	1.82	1084.4
C19	0.522	0.281	1.86	1044.7
C20	0.529	0.288	1.84	1058.5
C22	0.512	0.263	1.95	972.9
C23	0.512	0.268	1.91	993.5
C24	0.557	0.298	1.87	1128.6
C25	0.568	0.301	1.89	1137.8
C26	0.532	0.276	1.93	1017.3
C27	0.563	0.298	1.89	1034.5
C28	0.515	0.274	1.88	1038.2
C29	0.538	0.280	1.92	1059.9
C30	0.503	0.268	1.88	1169.3
C31	0.602	0.329	1.83	1136.2
C32	0.566	0.298	1.90	1076.3
C33	0.583	0.307	1.90	1164.2
C34	0.512	0.278	1.84	988.5
C35	0.532	0.281	1.89	1076.1

C36	0.545	0.292	1.87	1068.3
C37	0.502	0.265	1.89	978.1
C38	0.511	0.267	1.91	1063.6
C39	0.563	0.297	1.90	104I.2
C40	0.525	0.278	1.89	1089.4
C41	0.532	0.284	1.87	1075.3
C42	0.547	0.292	1.87	1095.5

Table S2. The 260/280 absorbance ratios falling within the range of 1.8 to 2.0 indicated that the RNA was free of contamination.

Table S3

Main parameters of singleplex PCR performed on p38 and β-actin

Sample No.	Target Name	Reporter	Ct Threshold	Baseline Start	Baseline End	Tm
C1	rat P38	SYBR	472.910875	1	37	79.32863617
C1	rat β-actin	SYBR	472.910875	1	37	70.67072296
C10	rat P38	SYBR	472.910875	1	37	77.39044952
C10	rat β-actin	SYBR	472.910875	1	37	70.52627563
C11	rat P38	SYBR	472.910875	3	37	69.63095093
C11	rat β-actin	SYBR	472.910875	3	37	69.63095093
C12	rat P38	SYBR	472.910875	1	37	67.0941925
C12	rat β-actin	SYBR	472.910875	1	37	74.10758209
C13	rat P38	SYBR	472.910875	1	37	70.67549896
C13	rat β-actin	SYBR	472.910875	1	37	66.49730682
C14	rat P38	SYBR	472.910875	1	37	82.46396637
C14	rat β-actin	SYBR	472.910875	1	37	68.73562622
C15	rat P38	SYBR	472.910875	3	37	71.86927032
C15	rat β-actin	SYBR	472.910875	1	37	77.68888855
C16	rat P38	SYBR	472.910875	3	37	73.21225739
C16	rat β-actin	SYBR	472.910875	1	37	72.01848602
C17	rat P38	SYBR	472.910875	1	37	65.75119781
C17	rat β-actin	SYBR	472.910875	1	37	72.46615601
C18	rat P38	SYBR	472.910875	1	37	75.30162048
C18	rat β-actin	SYBR	472.910875	3	37	73.51081848
C19	rat P38	SYBR	472.910875	1	37	80.67403412
C19	rat β-actin	SYBR	472.910875	3	37	79.9278717
C2	rat P38	SYBR	472.910875	3	37	65.74466705
C2	rat β-actin	SYBR	472.910875	3	37	66.64031219
C20	rat P38	SYBR	472.910875	1	37	65.45220184
C20	rat β-actin	SYBR	472.910875	1	37	80.82327271
C21	rat P38	SYBR	472.910875	1	37	66.34760284
C21	rat β-actin	SYBR	472.910875	1	37	73.80928802
C22	rat P38	SYBR	472.910875	3	37	69.03380585
C22	rat β-actin	SYBR	472.910875	1	37	76.79396057
C3	rat P38	SYBR	472.910875	1	37	77.68662262
C3	rat β-actin	SYBR	472.910875	3	37	78.13444519
C4	rat P38	SYBR	472.910875	1	37	66.34176636
C4	rat β-actin	SYBR	472.910875	1	37	71.11854553
C5	rat P38	SYBR	472.910875	1	37	77.53734589
C5	rat β-actin	SYBR	472.910875	3	37	67.98378754
C7	rat P38	SYBR	472.910875	1	37	90.22566986
C7	rat β-actin	SYBR	472.910875	1	37	71.26782227
C8	rat P38	SYBR	472.910875	3	37	71.71564484

C8	rat β-actin	SYBR	472.910875	1	37	78.58226776
C9	rat P38	SYBR	472.910875	1	37	66.49104309
C9	rat β-actin	SYBR	472.910875	1	37	71.26782227
C31	rat P38	SYBR	472.910875	1	37	67.94058882
C31	rat β-actin	SYBR	472.910875	3	37	68.11938588
C32	rat P38	SYBR	472.910875	1	37	72.00937478
C32	rat β-actin	SYBR	472.910875	1	37	81.11093757
C33	rat P38	SYBR	472.910875	1	37	66.29845869
C33	rat β-actin	SYBR	472.910875	1	37	70.83729477
C34	rat P38	SYBR	472.910875	1	37	83.42857468
C34	rat β-actin	SYBR	472.910875	1	37	69.18836547
C35	rat P38	SYBR	472.910875	3	37	69.38927648
C35	rat β-actin	SYBR	472.910875	1	37	75.29840006
C36	rat P38	SYBR	472.910875	3	37	72.38761938
C36	rat β-actin	SYBR	472.910875	1	37	74.48820585
C37	rat P38	SYBR	472.910875	1	37	67.58903726
C37	rat β-actin	SYBR	472.910875	1	37	73.28593019
C38	rat P38	SYBR	472.910875	1	37	72.1874619
C38	rat β-actin	SYBR	472.910875	3	37	78.39856101
C39	rat P38	SYBR	472.910875	1	37	81.26153341
C39	rat β-actin	SYBR	472.910875	3	37	84.93756256
C23	rat P38	SYBR	472.910875	1	37	69.22784669
C23	rat β-actin	SYBR	472.910875	1	37	76.27583658
C40	rat P38	SYBR	472.910875	1	37	68.38662861
C40	rat β-actin	SYBR	472.910875	1	37	83.2749178
C41	rat P38	SYBR	472.910875	1	37	69.37597149
C41	rat β-actin	SYBR	472.910875	1	37	76.82664917
C42	rat P38	SYBR	472.910875	3	37	67.13756947
C42	rat β-actin	SYBR	472.910875	1	37	78.47193765
C24	rat P38	SYBR	472.910875	1	37	79.68661262
C24	rat β-actin	SYBR	472.910875	3	37	72.27186638
C25	rat P38	SYBR	472.910875	1	37	70.38745535
C25	rat β-actin	SYBR	472.910875	1	37	85.83224766
C26	rat P38	SYBR	472.910875	1	37	76.48188985
C26	rat β-actin	SYBR	472.910875	3	37	83.948574
C27	rat P38	SYBR	472.910875	1	37	89.26336476
C27	rat β-actin	SYBR	472.910875	1	37	72.31150649
C28	rat P38	SYBR	472.910875	1	37	74.31829949
C28	rat β-actin	SYBR	472.910875	1	37	79.85938299
C29	rat P38	SYBR	472.910875	1	37	68.39857739
C29	rat β-actin	SYBR	472.910875	1	37	67.2172839

Table S3. By detecting DNA expression of p38 and β-actin, the samples were assured of not being contaminated with genomic DNA. Tm, melting temperature.
