# Supplementary figures and images for: The Role of p38 Mitogen-Activated Protein Kinase-Mediated F-Actin in the Acupuncture-Induced Mitigation of Inflammatory Pain in Arthritic Rats
Source: Brain Sci. 2024 Apr 14;14(4):380. doi: 10.3390/brainsci14040380 (PMC11048453; doi:10.3390/brainsci14040380)

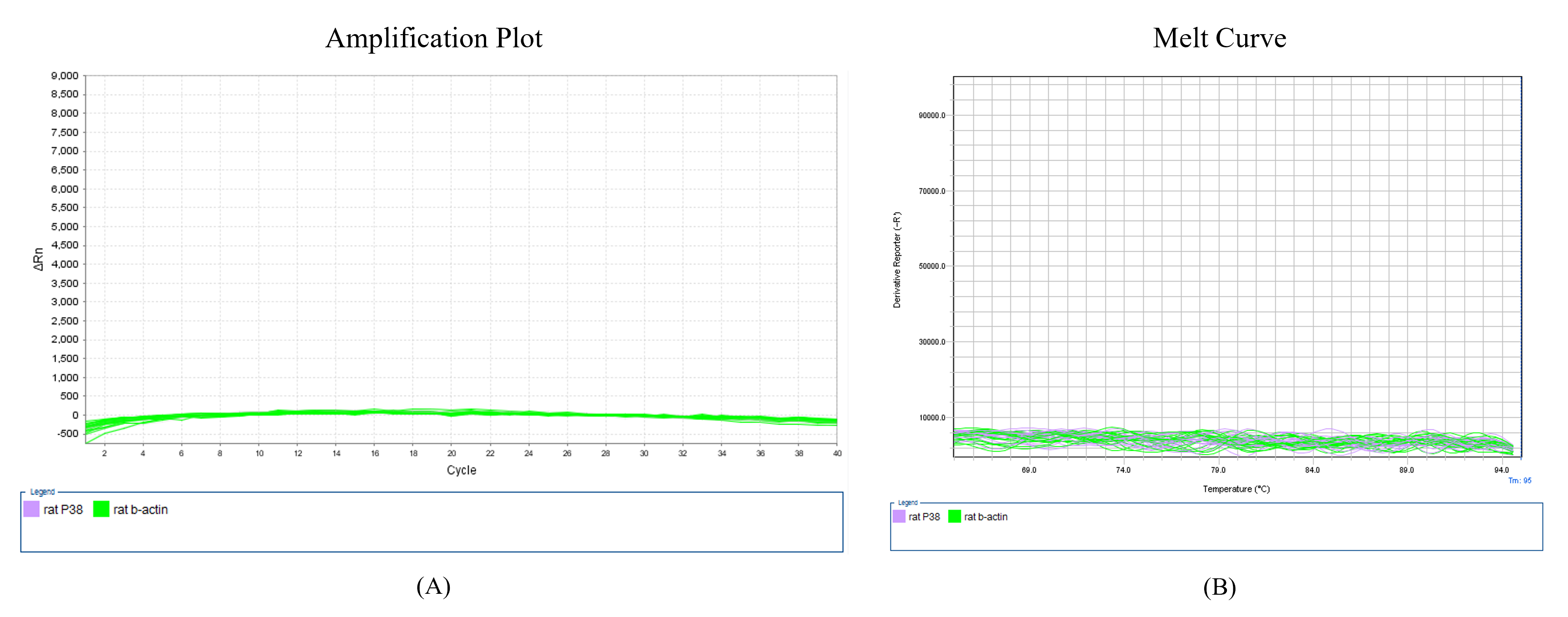

Supplement: Supplementary file 1 [file brainsci-14-00380-s001.zip › Figure S1.tif]

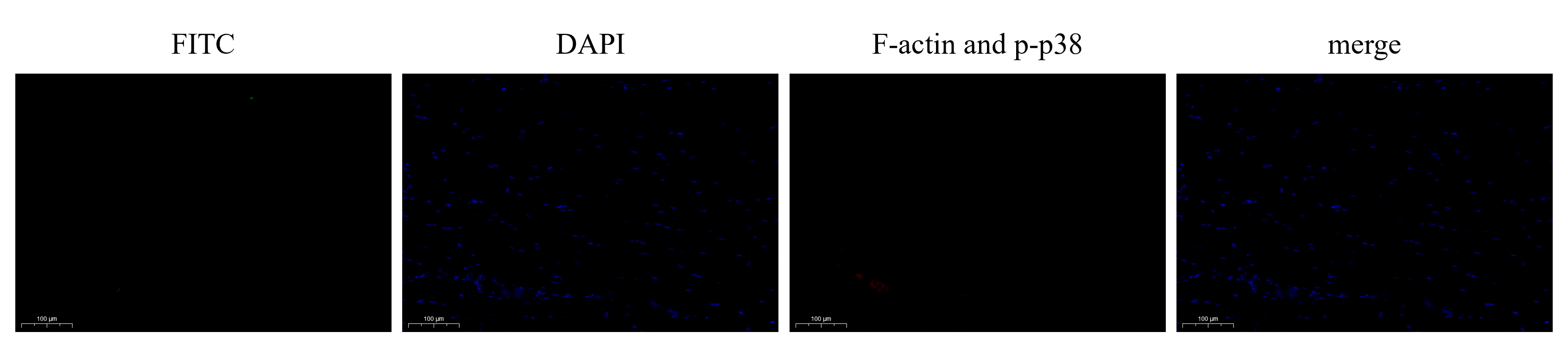

Supplement: Supplementary file 1 [file brainsci-14-00380-s001.zip › Figure S2.tif]

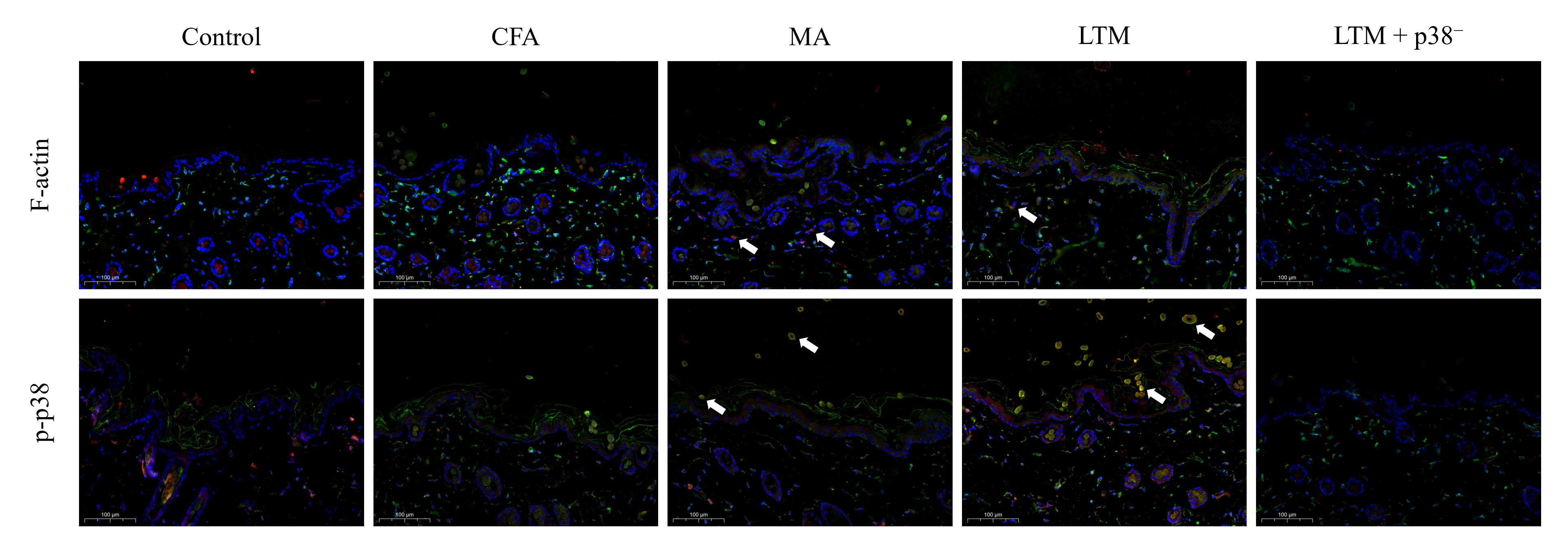

Supplement: Supplementary file 1 [file brainsci-14-00380-s001.zip › Figure S3.tif]
